# The EpiNo® Device: Efficacy, Tolerability, and Impact on Pelvic Floor—Implications for Future Research

**DOI:** 10.1155/2016/3818240

**Published:** 2016-02-14

**Authors:** Tilemachos Kavvadias, Irene Hoesli

**Affiliations:** Department of Obstetrics and Gynecology, University Hospital of Basel, Spitalstrasse 21, 4031 Basel, Switzerland

## Abstract

*Aims.* The aim of this review is to provide a comprehensive overview of the available literature on preventing perineal trauma with the EpiNo.* Methods*. We perfomed a literature research in the MedLine and EMBASE databases for studies referring to EpiNo published between 1990 and 2014, without restrictions for language and study type.* Results.* Five published studies were identified, regarding the effect of EpiNo on the rate of episiotomy and perineal tears, pelvic floor muscle function, and fetal outcome. The device seems to reduce episiotomy and perineal tears' rate, as well as the risk for levator ani microtrauma and avulsion, though not always statistically significant. It does not seem to have an effect on duration of second stage of labour and fetal outcome. The device is well tolerated and the adverse events are rare and mild. However, design and reporting bias in the reviewed articles do not allow evidence based conclusions.* Conclusions*. The EpiNo device seems to be promising, with potential positive effects on natural birth, while being uncomplicated to use and without major complications. Well designed, randomized trials are needed in order to understand the effects of EpiNo on pelvic floor and make evidence based recommendations on its use.

## 1. Introduction

Perineal injury is the most common maternal obstetrical complication associated with vaginal delivery [1]. It has been estimated that first- and second-degree perineal tears occur in 38% of spontaneous vaginal deliveries in primigravidae and in 36% in multiparae [2]. Perineal trauma is associated with significant maternal morbidity, including pain, dyspareunia, and physical and psychological impairment [3, 4]. The majority of perineal tears are of first and second degree but high-degree perineal injuries appear in 0.5% to 7% of vaginal deliveries [5]. Women who suffered anal sphincter trauma are in high risk of developing short- and long-term anal symptoms, such as fecal urgency or flatus and fecal incontinence [6].

Episiotomy is the most common obstetrical intrapartum intervention; episiotomy rates vary widely around the world and have been reported to be as low as 9.7% in Sweden, 46% in Switzerland, and up to 100% in Taiwan [7, 8]. Although episiotomy is still being performed routinely in some institutions, there is no evidence that it can prevent perineal damage, pelvic floor relaxation, and urinary or anal incontinence [9]. Additionally, published data suggest that episiotomy, when compared to spontaneous perineal lacerations, is associated with lower pelvic floor strength and higher rates of dyspareunia and perineal pain [10].

The morbidity following perineal trauma and episiotomy and the demand to optimize the fetomaternal health care have led to efforts to prevent perineal trauma and reduce the use of episiotomy. In particular, prevention, correct diagnosis, and management of high-degree perineal tears have been used as a quality marker for obstetrical units; the Royal College of Obstetrics and Gynecology devised in 2008 the “maternity dashboard,” a tool designed for use in the UK to benchmark and improve obstetric care, using high-degree perineal tears as quality indicators [11]. The World Health Organization refers to perineal protection during the second stage of labour as being an important contributor to high quality care-giving during birth and recommends the restricted use of episiotomy since there is no reliable evidence that its liberal use has a beneficial effect [12]. However, there is very limited data to date on effective measures against perineal trauma during childbirth. Various published studies suggest antenatal perineal massage, warm compresses, use of birth pools, and avoidance of the upright position [13–16]. A Cochrane review published in 2011 including 11.651 women showed that only the use of warm compresses during the second stage of labour could significantly reduce third- and fourth-degree tears (risk ratio (RR) 0.48, 95% confidence interval (CI) 0.28 to 0.84) [17].

In the beginning of 2000 a newly developed device appeared, called EpiNo. The manufacturer company (Tescana Munich, Germany) claims that EpiNo, just like the old African “calabash/gourd,” can facilitate a natural birth and reduce the risk of perineal injury, when inserted into the vagina in order to stretch the pelvic floor muscles [18]. Indeed, the first reports on EpiNo showed promising results [19]. However, more than ten years after its development, literature data is still sparse and no published article which would summarize published studies on EpiNo was identified. The aim of this review is to provide a comprehensive overview of the available literature on the way EpiNo affects perineal trauma and episiotomy rates, as well as its tolerability and safety.

## 2. Materials and Methods

A literature research in the MedLine and EMBASE databases for studies referring to EpiNo published between 1990 and 2014 was performed, without any language and study type restrictions.

At the time of writing, five published studies could be identified regarding the effects of EpiNo on the rate of episiotomy and perineal tears, pelvic floor muscle function, and fetal outcome (Table 1).

## 3. Results

### 3.1. Episiotomy and Perineal Tears

Two randomized control trials found no significant differences in the episiotomy rates between EpiNo users and controls [20, 21]. Ruckhäberle et al. reported a tendency for less episiotomy in the EpiNo group (41.9 versus 50.5%, *p* = 0.11). Shek et al. however performed overall less episiotomies, but more in the EpiNo group (29% versus 22%). This difference was not statistically significant (*p* = 0.40) though. The study groups in the two randomized clinical trials of Ruckhäberle and Shek had similar demographics. The difference in the episiotomy rates in the two trials could be a result of different obstetrical management in the two institutions. Both authors do not clarify either the episiotomy indications in their institutions or whether the episiotomy is performed routinely, sporadically, or restricted. Kovacs et al., in an observational study, did not find any significant differences in episiotomy rates [22]. Hillebrenner et al. reported a significant reduction in the episiotomy rate of 43% (OR 0.21); their study was a rather small cohort/observational study (fifty pregnancies) with retrospective matched pair comparison of EpiNo users and controls [23]. Kok et al. reported a massive reduction of episiotomy in an institution where episiotomy was routinely performed (50% versus 93.3%, *p* < 0.0001) [24].

Results on perineal tears are controversial. Again the results of Shek and Ruckhäberle are inconsistent. Shek et al. found no differences in perineal tear rate, while Ruckhäberle reported that an intact perineum is more frequent in the EpiNo group (37.4% versus 25.7%, *p* = 0.05). The work of Ruckhäberle was a multicentre trial and, as stated by the authors, obstetrical manoeuvres or techniques routinely used for the perineal protection in different institutions were not reported. There was also no stratification of the results of each institution, and their results are thus difficult to evaluate [25, 26]. Other studies did not show any statistically significant differences in perineal tear rates between EpiNo users and controls. Kok et al. reported a reduction in the severity of perineal trauma, which was however not statistically significant [24].

### 3.2. Second Stage of Labour, Analgesics, and Fetal Outcome

Hillebrenner et al. reported a significant shorter 2nd stage of labour in EpiNo users (29 ± 25 minutes versus 55 ± 54 minutes, *p* = 0.014). They also found that women in the EpiNo group made use of less opioids intrapartum (15.8 versus 42.1%, *p* = 0.03) and asked less frequently for an epidural anaesthesia (15.6 versus 35.6%, *p* = 0.03) [27]. Other studies could not reproduce these results.

Fetal outcome and APGAR score seem not to be affected by the use of EpiNo, since none of the published studies could show significant differences, with the exception of Hillebrenner et al., who reported higher one-minute APGAR scores in the EpiNo group [23].

### 3.3. Impact on Pelvic Floor Muscle

Shek et al. published a randomized controlled trial in which levator ani avulsion and microtrauma during birth were assessed and compared among eighty-one EpiNo users and sixty-four controls, all primiparae. Their assessment included a 4-dimensional translabial ultrasound in the 33rd–35th week of gestation and 3 months postpartum. Levator avulsion and microtraumata were diagnosed using a standardised method of measuring the levator volume and the hiatal area [20]. Shek and Dietz found that although EpiNo users did not show any differences in episiotomy rate and perineal tears, they did have a lower, but not statistically significant, risk of levator ani avulsion (12% versus 7%, RR 0.62 (CI 0.22–1.76), *p* = 0.37) and microtrauma (30% versus 21%, RR 0.68 (CI 0.37–1.25), *p* = 0.22) [25]. Dietz et al., in the 44th Annual Meeting of the International Continence Society, presented unpublished results of a large multicentre randomized control trial, including 335 EpiNo users and 325 controls, and reported that EpiNo could not provide any protective effect on the external anal sphincter but also had no negative effect on pelvic floor function [26].

### 3.4. Tolerance, Compliance, and Complications

The recommended use in each trial can be seen in Table 1. Generally, the use of EpiNo seems to be well tolerated. Nakamura et al., in an observational study on perineal distensibility tolerance, included 227 parturient women who were first time introduced to the EpiNo on admission to the labour ward and measured the maximal circumference reach of the EpiNo during dilatation period [28]. Results showed a mean circumference of 19.6 cm (SD 2.7 cm) and mean visual analogue scale pain score of 3.8 cm (SD 2.6 cm). The correlation between maximal circumference and pain score was only fair (Spearman's *r* = 0.424), which could reflect the variable individual pain thresholds. Ruckhäberle et al. demonstrated a mean EpiNo dilatation of 24.3 cm (SD 4.4) after training [21]. Comparing the two trials, it is unclear whether the greater maximal dilatation in the trial of Ruckhäberle could be reached gradually, or EpiNo users were biased by the home measurement, or even whether an eventually stressful labour ward admission could lead to lower tolerance in the study of Nakamura.

There were no reported drop-outs in the use of EpiNo, but some compliance issues were reported. The recommended use duration was not always reached. Interestingly, Shek and Dietz [25] did find a correlation between usage time and levator ani microtrauma risk (38% to 26% and to 17% for women in the EpiNo group who did not use the device and used it ≤20 times and >20 times, resp., *p* = 0.40) [25]. Other studies did not mention the actual usage time.

Complications seem to be mild and of minor significance, without affecting the pregnancy and fetal outcome. Bleeding (8.2%), pain (8.9%), uterine contractions (1.5%), and dislocation of the device from the vagina (15.6%) have been reported [21, 22]. A single major complication was published by Nicolle and Skupski, a case of a young woman in 37 weeks of gestation who suffered near cardiovascular collapse while using the EpiNo and was admitted with symptoms of venous air embolism, although there was no evidence that the symptoms were associated with the use of the device [27].

## 4. Discussion

The first report of a birth canal dilator was published in 1991 by Hofmeyr and Bassin. They presented a cylindrical shaped device, which could be inserted into the vagina during labour and be inflated using saline to a maximum diameter of 10 cm. The principle of action was the controlled dilatation of the pelvic floor soft tissues, thus gradually preparing the birth canal for the fetal head, in contrast with the uncontrollable expulsive distension of the birth canal [29]. Although the authors reported a tendency for lower rates of assisted delivery, shorter duration of the second stage of labour, and lower pain scores postpartum, they were confronted with massive technical flaws of their patented device, which led to inconsistent distension.

The EpiNo seems to be technically of good quality. None of the published studies reported technical issues. The studies of Nakamura et al. on perineal distensibility [28] imply a good reproducibility of the distention effect. However, being a device designed for home use, it is prone to various biases, such as inconsistency in frequency and number of sessions.

Another issue of the reviewed papers is the design and performance bias. None of the studies report any concomitant factors during second stage of labour, which could have an effect on perineal trauma: other perineal techniques (such as warm compresses), birth position, and obstetrical manoeuvres are not mentioned. Also good designed studies, such as the ones of Heaberle et al. and Shek et al., suffer from low statistical power, which is important when interpreting the results. The study of Kok et al. was performed in an institution where routine episiotomy was common; Hillebrenner et al. used matched pairs comparison and control group data were obtained retrospectively. There is also no available data on patients' satisfaction and ease of use as well as long-term data on pelvic floor function, urinary and fecal incontinence, and dyspareunia after the use.

The EpiNo birth trainer seems to be a promising device, with positive effects on natural birth. In some countries it has already been a marketing success, since it appeals to future mothers, through its uncomplicated use, low complication profile, and practically absence of serious adverse events and the potential benefit on pelvic floor function. However the current literature still lacks high quality trials, which would meticulously investigate the effects of EpiNo. Some suggestions for future research could be to design randomized trials with good statistical power, avoid reporting and performance bias (obstetrical interventions and perineal techniques), standardise the use of EpiNo (frequency and number of sessions), assess patients' satisfaction, and obtain long-term data on pelvic floor function, dyspareunia, and urinary and fecal incontinence.

## Figures and Tables

**Table 1:** Published studies between 2001 and 2011 in chronological order, showing the study populations, sessions with the EpiNo , and outcomes.

Year of publication	Author	Type of study	Primary outcome	Inclusion criteria	Sessions	Study population	Follow-up	Results
2011	Shek et al. [20]	Prospective RCT Single blinded	Does EpiNo reduce levator ani trauma?	Singleton, age over 18, uncomplicated pregnancy	37 w to delivery, up to 2 × 20 minutes daily	81 EpiNo users, 64 controls	3 months after delivery	(a) Reduced risk for avulsion of levator ani (6% versus 13%, *p* = 0.19) (b) No difference in perineal tears and duration of second stage of labour

2009	Ruckhäberle et al. [21]	Multicenter prospective RCT Single blinded	Does EpiNo reduce perineal trauma?	Singleton, primigravid	37 1/7 to delivery, minimum of 15 minutes daily	107 EpiNo users, 15 controls	3 months after delivery	(a) Higher incidence of intact perineum (37,4% versus 25.7%, *p* = 0.05) (b) Tendency towards decreased episiotomy rate (41.9 versus 50.5%, *p* = 0.11) (c) No difference in duration of second stage of labour, APGAR scores, Oxford grading of pelvic muscles, bladder neck mobility, and infections rates

2004	Kovacs et al. [22]	Observational case-control	Effect of EpiNo on perineum	Primigravid	From 37 w to delivery, 15 minutes daily for 14 consecutive days	39 EpiNo users, 248 controls	Directly postpartum	(a) Intact perineum in 46% versus 17% (*p* < 0.0001), perineal tears 28% versus 49% (*p* < 0.05), episiotomy 26% versus 34% (*p* = 0.286) (b) No difference in second-stage duration, incidence of instrumental delivery, or APGAR scores

2004	Kok et al. [24]	Case-control (retrospective analysis of controls)	Episiotomy rate, perineal tear rate, and analgesic requirements postpartum	Primigravid, single pregnancy	36–38 weeks, 15 minutes daily for 14 days	20 EpiNo users, 60 controls	Directly postpartum	(a) Episiotomy 50% versus 93.3% (*p* < 0.0001) (b) Perineal trauma 90.0% versus 96.6%, *p* = 0.24

2001	Hillebrenner et al. [23]	Prospective observational single blinded case-control	Episiotomy and perineal tear rates, duration of second stage of labour, analgesic requirements, epidural anesthesia rates, and fetal outcome	Primigravid or after primary cesarean section, no infection	From 38 0/7 to delivery, 10 minutes daily	45 EpiNo users, 45 controls	Directly postpartum	(a) Episiotomy 82% versus 47% (*p* < 0.001) (b) No significance in perineal tears (*p* = 0.4) (c) Intact perineum 9 versus 49% (d) Higher 1-minute APGAR scores (*p* = 0.024) (e) Second-stage duration 29 ± 25 min versus 55 ± 54 min (*p* = 0.014) (f) Less epidural (15.6% versus 35.6%, *p* = 0.03)
